# Piperine improves levodopa availability in the 6‐OHDA‐lesioned rat model of Parkinson's disease by suppressing gut bacterial tyrosine decarboxylase

**DOI:** 10.1111/cns.14383

**Published:** 2023-08-01

**Authors:** Xiaolu Hu, Lan Yu, Yatong Li, Xiaoxi Li, Yimeng Zhao, Lijuan Xiong, Jiaxuan Ai, Qijun Chen, Xing Wang, Xiaoqing Chen, Yinying Ba, Yaonan Wang, Xia Wu

**Affiliations:** ^1^ Beijing Key Lab of TCM Collateral Disease Theory Research, School of Traditional Chinese Medicine Capital Medical University Beijing China; ^2^ Department of Pharmacy Xuanwu Hospital of Capital Medical University Beijing China; ^3^ School of Pharmaceutical Sciences Capital Medical University Beijing China; ^4^ Core facilities of modern pharmaceuticals Capital Medical University Beijing China

**Keywords:** *Enterococcus faecalis*, levodopa, Parkinson's disease, piperine, tyrosine decarboxylase

## Abstract

**Aim:**

Tyrosine decarboxylase (TDC) presented in the gut‐associated strain *Enterococcus faecalis* can convert levodopa (L‐dopa) into dopamine (DA), and its increased abundance would potentially minimize the availability and efficacy of L‐dopa. However, the known human decarboxylase inhibitors are ineffective in this bacteria‐mediated conversion. This study aims to investigate the inhibition of piperine (PIP) on L‐dopa bacterial metabolism and evaluates the synergistic effect of PIP combined with L‐dopa on Parkinson's disease (PD).

**Methods:**

Metagenomics sequencing was adopted to determine the regulation of PIP on rat intestinal microbiota structure, especially on the relative abundance of *E. faecalis*. Then, the inhibitory effects of PIP on L‐dopa conversion and TDC expression of *E. faecalis* were tested in vitro. We examined the synergetic effect of the combination of L‐dopa and PIP on 6‐hydroxydopamine (6‐OHDA)‐lesioned rats and tested the regulations of L‐dopa bioavailability and brain DA level by pharmacokinetics study and MALDI‐MS imaging. Finally, we evaluated the microbiota‐dependent improvement effect of PIP on L‐dopa availability using pseudo‐germ‐free and *E. faecalis*‐transplanted rats.

**Results:**

We found that PIP combined with L‐dopa could better ameliorate the move disorders of 6‐OHDA‐lesioned rats by remarkably improving L‐dopa availability and brain DA level than L‐dopa alone, which was associated with the effect of PIP on suppressing the bacterial decarboxylation of L‐dopa via effectively downregulating the abnormal high abundances of *E. faecalis* and TDC in 6‐OHDA‐lesioned rats.

**Conclusion:**

Oral administration of L‐dopa combined with PIP can improve L‐dopa availability and brain DA level in 6‐OHDA‐lesioned rats by suppressing intestinal bacterial TDC.

## Graphical abstract

The combination of piperine and L‐dopa can better ameliorate the motor disorders of 6‐OHDA‐lesioned rats by improving the availability of oral L‐dopa and brain DA levels through suppressing the intestinal bacterial TDC‐dependent L‐dopa metabolism.

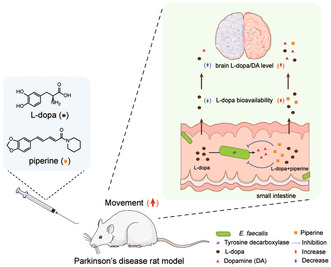

## 1 INTRODUCTION

Parkinson's disease (PD) is a common age‐related neurodegenerative disorder affecting more than 1% of global population over the age of 60,^1^ and the incident rate keeps increasing with the aging of the population, which will lead to certain social pressures due to the surging demand for productivity and medical resources.^2^, ^3^ PD patients are characterized by the progressive loss of dopaminergic neurons, which results in the lack of dopamine (DA) in basal ganglia.^4^ Therefore, discovering safe and effective therapeutics to elevate the brain DA level in PD patients is one of the main strategies of PD treatments.^5^

Levodopa (L‐dopa), the precursor of DA, is the primary treatment of PD.^6^ To ensure that the brain DA can be adequately supplemented, L‐dopa is currently used in combination with an aromatic amino acid decarboxylase inhibitor (AADCI) to decrease its extensive peripheral metabolism.^7^, ^8^ However, the effect of the commonly used inhibitor is limited. When combined with carbidopa (AADCI), nearly 56% of L‐dopa is still metabolized and the treatment outcomes are varied significantly among PD patients.^9^ Particularly a subset of PD patients with low efficacy^10^ need more frequent drug treatment over time, which can lead to higher risks of dyskinesia and other side effects like constipation, cardiac arrhythmias, and orthostatic hypotension.^11^

Gut microbiota metabolism is now considered to be one of the key factors interfering with the effectiveness of drug treatment.^12^, ^13^ Recent studies have demonstrated that oral L‐dopa could be metabolized by gut bacteria. It is found that a pyridoxal‐5′‐phosphate (PLP)‐dependent tyrosine decarboxylase (TDC) mainly encoded by the gut‐associated strain *Enterococcus faecalis* has strong decarboxylase activity toward L‐dopa.^14^ Its abundance can limit the bioavailability of L‐dopa and lead to the highly inter‐individual variation in efficacy. Now the host target inhibitors used in clinic hardly prevent L‐dopa conversion by bacterial TDC,^15^ so it will be a significant and potentially innovative way for PD adjuvant therapy to find a compound that can effectively inhibit the activity of intestinal bacterial TDC.

**FIGURE 1:**
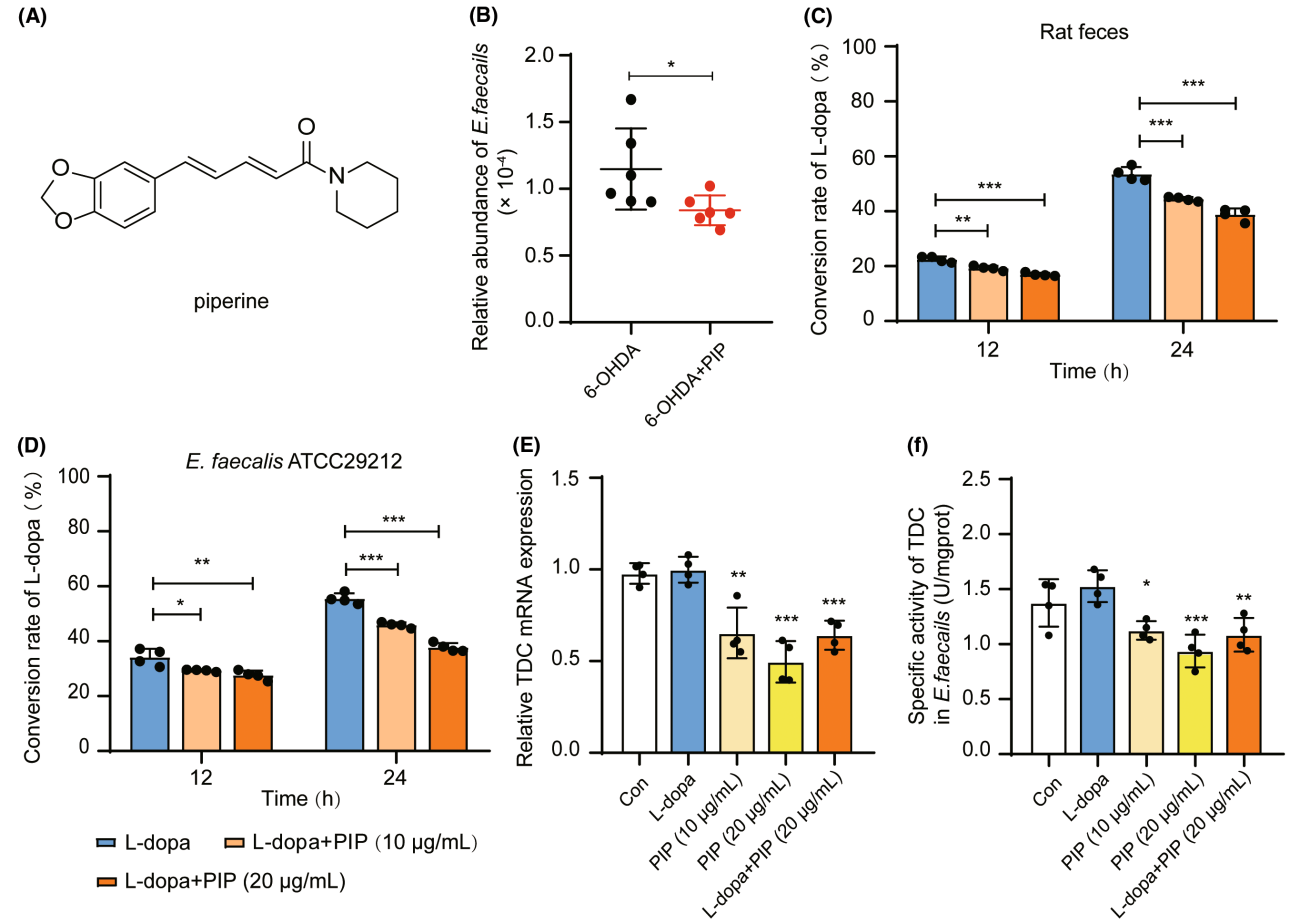
PIP inhibited intestinal bacterial L‐dopa conversion in vitro. (A) The chemical structure of PIP. (B) The relative abundance of *E. faecalis* in 6‐OHDA‐lesioned rats with PIP administration. The 6‐OHDA‐lesioned rats were treated with PIP (20 mg/kg) or the same volume of substrate solution (0.5% CMC‐Na) once a day for 6 weeks, and then colonic contents were collected for metagenomics analysis (*n* = 6). (C) The conversion rate (CR) of L‐dopa in the rat feces culture in vitro. Rat feces were incubated with L‐dopa (0.3 mM) and L‐dopa (0.3 mM) + PIP (10 or 20 μg/mL) for 12 and 24 h, respectively. The levels of remaining L‐dopa in systems were determined by HPLC‐FLD. The CR is calculated according to the formula: “CR = |*C*_t_ – *C*_I_|/*C*_I_ × 100%” (*C*_t_: remaining concentration of L‐dopa at t h; *C*_I_: initial concentration of L‐dopa). (D) The CR of L‐dopa in *E. faecalis* ATCC29212 culture. The cultures of *E. faecalis* ATCC29212 together with L‐dopa (0.3 mM) and various concentrations of PIP (10 and 20 μg/mL) were incubated for 12 and 24 h under anaerobic conditions, and the contents of L‐dopa were detected by HPLC‐FLD. The relative gene expression (E) and specific activity (F) of TDC in *E. faecalis*. *E. faecalis* ATCC29212 was co‐incubated with L‐dopa (0.3 mM), PIP (10 μg/mL), PIP (20 μg/mL), and L‐dopa (0.3 mM) + PIP (20 μg/mL) for 24 h, respectively. The control group was treated with solvent (2% ethanol). The relative expression of TDC mRNA was determined by RT–qPCR. The specific activity of TDC in *E. faecalis* was tested by sandwich ELISA. Data are presented as mean ± SD (*n* = 4). *p < 0.05 versus 6‐OHDA group (Student's *t*‐test); *p < 0.05, **p < 0.01, ***p < 0.001 versus L‐dopa group (one‐way ANOVA followed by Tukey's multiple‐comparison test).

In our study, we occasionally found that the relative abundance of *E. faecalis* was significantly decreased in the 6‐hydroxydopamine (6‐OHDA)‐lesioned rats after piperine (PIP, Figure 1A) administration by the metagenomics analysis of gut microbiota (Table S1). PIP is the major component of *Piper longum* L., a medicine food homology herb to treat pain, vomiting, and diarrhea in traditional Chinese medicine (TCM), and the total alkaloids of *P. longum* (PLA) contain more than 50% PIP.^16^ We have proved that PIP and PLA exerted neuroprotective effects on 6‐OHDA, lipopolysaccharide (LPS), and 1‐methyl‐4‐phenyl‐1,2,3,6‐tetrahydropyridine (MPTP)‐induced PD rat or mice models, which could alleviate motor deficits and improve the brain DA level.^17^, ^18^, ^19^ However, it is unclear whether the regulation of *E. faecalis* by PIP plays a crucial role in increasing the brain DA level, especially when PIP is combined with L‐dopa during PD treatment. These provoked our interest in exploring the synergistic effect of PIP combined with L‐dopa to increase DA. We hypothesized that PIP may enhance the effectiveness of L‐dopa on PD model rats by improving the L‐dopa availability and brain DA level through interfering with the intestinal bacterial decarboxylation of L‐dopa.

In the present study, we explored the mechanism of PIP in suppressing L‐dopa metabolism of *E. faecalis*, and demonstrated that the combination of PIP and L‐dopa can better ameliorate the motor disorders of 6‐OHDA‐lesioned rats than L‐dopa alone by improving the availability of oral L‐dopa and brain DA level. In addition, we further evaluated the improvement effect of PIP on L‐dopa availability in pseudo‐germ‐free (PGF) and *E. faecalis*‐transplanted rats to confirm this was crucially related to the suppressive effect of PIP on intestinal bacterial L‐dopa metabolism. The results provide a new idea and scientific support for the treatment of PD with integrated traditional Chinese and Western medicine.

## 2 MATERIALS AND METHODS

### 2.1 Chemicals

PIP with purity ≥ 99% was purchased from Chengdu Push Biotechnology Co., Ltd. L‐dopa (H31020888) and Madopar (L‐dopa/benserazide 4:1, H10930198) used in rat administration were produced by Shanghai Fuda Pharmaceutical Co., Ltd., and Shanghai Roche Pharmaceutical Co., Ltd, respectively.

### 2.2 Animals

Sprague–Dawley rats (180–200 g) were purchased from Beijing Charles River Lab Animal Technology Co., Ltd., then housed in the SPF environment under standard conditions (temperature: 25°C ± 2°C, humidity: 50% ± 15%, a 12 h light/dark cycle, and adequate water and food). All experimental procedures were approved by the Animal Experiments and Experimental Animal Welfare Committee of Capital Medical University, and the ethics approval number of this study is AEEI‐2015‐082.

### 2.3 PD rat models and treatments

Adaptively raised for 1 week, SD rats were randomly divided into two groups: the sham‐operated group (*n* = 6) and the 6‐OHDA‐lesioned group (*n* = 36). After deep anesthesia, the rats were stereotaxically injected with 3 μL 6‐OHDA (4 mg/mL in 0.02% ascorbic acid saline; Sigma) into the left striatum following our previously described protocol,^17^ and the rats in sham group were injected with 3 μL vehicle (0.02% ascorbic acid saline).

The damage of rat dopaminergic neurons was assessed by apomorphine (APO, Sigma)‐induced rotation tests at the 5th, 8th, and 11th weeks of experiment.^17^ At the end of the test on the 5th week, the 6‐OHDA‐lesioned rats were randomly divided into five treatment groups: (1) 6‐OHDA + L‐dopa (60 mg/kg) group,^20^ (2) 6‐OHDA + PIP (10 mg/kg) group, (3) 6‐OHDA + PIP (20 mg/kg) group, (4) 6‐OHDA + L‐dopa (60 mg/kg) + PIP (20 mg/kg) group, and (5) 6‐OHDA + madopar (75 mg/kg) group. The dosages of PIP used in the present study were safe for rats.^21^ Rats were treated once a day for 6 weeks. The 6‐OHDA group and sham group were treated with the same volume of substrate solution (0.5% CMC‐Na).

### 2.4 Behavior tests

Open‐field test was conducted on the 2nd day after the rotation test. The movements in 5 min of rats in open‐field apparatus (100 × 100 × 40 cm), including total movement distance (cm) and horizontal velocity (cm/s), were recorded by the TruScan Activity Monitoring System (USA). Abnormal involuntary movements (AIMs) were scored according to the rating system described in Lee's study.^22^ Briefly, rats were habituated in single cages for 30 min before gavage. Then, the dyskinesia behaviors of rats, including axial dystonia, reverse rotation, and limb and masticatory dyskinesia, were evaluated during a 1‐min monitoring period, repeated every 20 min for 2 h. Each subtype was scored from 0 to 4 according to severity (0, absent; 1, occasional; 2, frequent; 3, continuous; and 4, continuous and not disturbed by external stimuli), and the AIMs of rats were assessed weekly during the treatment.

### 2.5 Western blot assay

The left substantia nigra (SN) of rat was lysed using non‐denaturing lysis buffer, and immunoblotting was performed following the reported protocol.^19^ Protein homogenates were subjected to Western blot analysis using antibodies against tyrosine hydroxylase (TH, 1:1000, Sigma) and GAPDH (1:2000, Proteintech). The blots were visualized by a NcmECL Ultra Western blot diction kit (NCM Biotech), and densitometry was analyzed by using the Fusion FX Image system (Vilber Lourmat).

### 2.6 Quantitative analysis of L‐dopa by HPLC‐FLD

The determinations of L‐dopa in biological samples were carried out using a Waters e2695 liquid chromatography system with a fluorescence detector (2475, Waters) equipped with a Waters Atlantis® T3 column (150 × 4.6 mm, 5 μm). The mobile‐phase system was composed of 10 mM ammonium format aqueous solution (A) and methanol (B). The HPLC elution conditions were as follows: 0–3 min, 1% B, 3–4 min, 1%–10% B, 4–8 min, 10% B, 8–9 min, 10%–95% B, 9–14 min, 95% B, and 14–15 min 95%–1% B. The flow rate was 1 mL/min, and the injection volume was 10 μL. The column was maintained at 40°C. The detector was set up for an excitation wavelength of 312 nm and an emission wavelength of 278 nm.

### 2.7 Pharmacokinetic analysis of L‐dopa

After fasted for 12 h, blood samples of rats were collected into heparinized tubes at various time points of 0, 0.25, 0.5, 1, 2, 3, 4, 6, and 10 h after dosing. After centrifugation at 1000 *g* (4°C) for 20 min, 50 μL plasma was separated and mixed with 400 μL of acetonitrile containing 2% formic acid and 0.5% sodium pyrosulfite, then vortexed for 5 min to precipitate proteins.^23^ After centrifugation at 12,000 *g* (4°C) for 20 min, 350 μL of supernatant was dried under the N_2_ atmosphere and re‐dissolved in 100 μL of 0.2% formic acid aqueous solution. After centrifugation again, the supernatant was obtained for HPLC‐FLD analysis.

### 2.8 Quantitation of striatal L‐dopa and DA

Rat striatum tissue was weighed and homogenized in 0.4 M perchloric acid solution, then incubated in ice for 1 h. After centrifugation at 12,000 *g* (4°C) for 20 min, the supernatant was collected and mixed with the buffer solution (2:1, V/V; containing 20 mM potassium citrate, 300 mm dipotassium hydrogen phosphate, and 2 mM EDTA · 2Na), then incubated in ice for another hour. After centrifugation, the supernatant was obtained and used for the determination of L‐dopa by HPLC‐FLD, and the content of DA was analyzed by HPLC‐ECD. The HPLC‐ECD conditions were set as described in the previous work.^18^ Briefly, the Waters e2695 liquid chromatography system with electrochemical detector (Waters 2465) equipped with a Waters Sunfire® C18 column (150 × 4.6 mm, 5 μm) was used for separation. The mobile phase consisted of buffer solution (containing 50 mM sodium citrate, 0.1 mM EDTA · 2Na, and 0.2 mM 1‐octa nesulfonic acid sodium salt; A) and methanol (B). Binary isocratic elution at a flow rate of 0.8 mL/min (A : B = 92:8, V/V).

### 2.9 MALDI‐MS imaging of DA in rat brain

The MALDI‐MS imaging method followed Yang W's description^24^ with some modifications. The brain tissues of rats were isolated 1 h after treatment and frozen in liquid nitrogen immediately, then embedded in 2% CMC‐Na and frozen overnight at −80°C. Striatum sections (20 μm) were prepared by the cryostat microtome (Leica CM1950), then transferred onto conductive ITO glass slides (Bruker) and stored at −80°C until analysis. 2,4,6‐Trimethylpyrylium tetrafluoroborate (TMP‐TFB, Aladdin) was used as the derivatization reagent (7.6 mg/mL in methanol buffered with 3.5 μL of trimethylamine), and α‐cyano‐4‐hydroxycinnamic acid (CHCA, Aladdin; 10 mg/mL, prepared in 70% methanol solution containing 0.1% trifluoroacetic acid) was used as the MALDI matrix. MALDI‐MS imaging experiments were performed on the MALDI‐FT‐ICR mass spectrometer (Solarix 9.4 T, Bruker) in positive‐ion mode. Set the pixel size of the imaging resolution at 80 nm to obtain the optimal acquisition performance. MS imaging data were visualized using Fleximaging (Bruker). After the data were normalized by the total ion current, the DA mass spectrum peak (*m/z* 258.1502 ± 0.002) was determined. The colored spots in the image represent the location of the neurotransmitter, and the color of each spot is related to the signal intensity detected by the laser at each pixel.

### 2.10 Measurement of L‐dopa conversion rate

The whole blood, colon contents, jejunum, and brain tissues of SD rats (*n* = 6) were collected. Colonic contents were pooled and suspended in anaerobic medium (Aobox) at a ratio of 1:20 (g/mL), the mixtures were centrifuged at 800 *g* (4°C) for 10 min, and then collected the supernatant for culture. Tissues were pooled and weighed, then homogenized with 5 mM phosphate buffer (pH = 7.4) at the ratio of 1:5 (g/ml). The incubation systems of rat intestinal bacteria, tissue homogenate, and whole blood were treated with L‐dopa (0.3 mM, Sigma) and L‐dopa (0.3 mM) + PIP (10 or 20 μg/mL), respectively. The intestinal bacterial cultures were incubated in an anaerobic chamber, while tissues or blood incubation systems were incubated at 37°C for 12 and 24 h, and then the incubation was terminated with three‐fold acetonitrile. After centrifugation at 12,000 *g* for 10 min, the supernatants were collected for HPLC‐FLD analysis.

### 2.11 Evaluation of the inhibitory effect of PIP toward TDC by enzyme assay

TDC (from *E. faecalis* NCTC6783, Aladdin; 0.02 mg/mL) and PLP (2 mM, Sigma) were pre‐incubated with various concentrations (0.01–250 μM) of PIP in 0.2 M sodium acetate buffer (pH = 5.5) at 37°C for 15 min. Then, the enzyme reaction was initiated by adding L‐dopa (final concentration was 0.3 mM). After 20 min of incubation, the reaction was quenched by diluting 1:10 in methanol containing 0.7% perchloric acid.^25^ The mixture was centrifuged at 12,000 *g* (4°C) for 5 min, and the supernatant was collected for the determination of DA by HPLC‐ECD.

### 2.12 Incubation of *Enterococcus faecalis* in vitro

*Enterococcus faecalis* ATCC 29212 was cultured overnight in tryptic soy broth (TSB, Solarbio), diluted in fresh broth to the final concentration of 1.0 × 10^6^ colony‐forming units (CFUs)/mL, and then incubated with PIP (10 or 20 μg/mL), L‐dopa (0.3 mM), and L‐dopa (0.3 mM) + PIP (10 or 20 μg/mL), respectively. The growth control group and negative control group were set. The cultures were incubated at 37°C under an anaerobic environment. The 1 mL of incubation sample and twofold cold acetonitrile were added to terminate the incubation. After centrifugation at 12,000 *g* for 10 min, the supernatants were collected for HPLC‐FLD analysis.

### 2.13 RT–qPCR assay

Total RNA was extracted from the culture pellets generated in the experiment described above “Incubation of *E. faecalis* in vitro” using the RNAprep Pure Cell/Bacteria Kit (TIANGEN). A FastQuant RT Kit (with gDNase) (TIANGEN) was used to transcribe RNA to cDNA. After that, RT–qPCR was conducted using 2 × SuperReal PreMix Plus (TIANGEN) and performed on the CFX Connect™ Real‐Time System (Bio–Rad) following the amplification program: 95°C for 15 min, followed by 40 cycles of 95°C for 10 s, 52°C for 31 s, and 72°C for 30 s. The program ended with a final extension at 72°C for 10 min. The primer sequences were listed in Table S2. The relative gene expression was calculated using the ${{2^{-}}^{\Delta \Delta }}^{C_{t}}$ method.

### 2.14 Determination of TDC in *Enterococcus faecalis* by ELISA

The bacterial protein was extracted from the culture pellets generated as described above. After being washed with PBS three times, the bacterial pellets were re‐suspended in 400 μL PBS containing 2% ethanol and incubated in ice bath for 30 min. After centrifugation at 1000 *g* for 10 min, the sediments were collected and lysed using lysis buffer containing 100 μg/mL lysozyme, then centrifuged at 12,000 *g* for 20 min to collect the supernatants. After determining the protein concentration using the BCA kit (Beyotime), the assay of TDC level in *E. faecalis* was performed according to the Microorganism TDC Elisa kit (MM‐925239O2, MEIMIAN) guideline. Briefly, the extracted protein samples were added to the coated wells together with the balance solution and HRP conjugate reagent, then incubated at 37°C. After washing the plate five times, solution A and solution B were added into the wells, and then incubated for 15 min. After adding the stop solution to each well, the optical density was measured at the wavelength of 450 nm. The specific activity of TDC was calculated based on the standard curve. The results are expressed as U/mg protein.

### 2.15 Metagenomics analysis

The gut microbiota of rats from 6‐OHDA group and 6‐OHDA + PIP (20 mg/kg) group were analyzed using metagenomics sequencing. The fecal microbial genomic DNA samples were extracted by QIAamp DNA stool mini kit (QIAGEN) following the manufacturer's instructions. Then, the fecal microbial genomic DNA samples were sent to the Beijing Institute of Genomics, Chinese Academy of Sciences, where metagenomics analysis was performed on an Illumina HiSeq2000 sequencing platform (Illumina Inc.) according to the protocol described in the published research.^26^ The species composition and abundance information of the samples were analyzed and counted.

### 2.16 Quantification of *Enterococcus faecalis* and TDC in feces

Fecal microbial genomic DNA was extracted using the TIANamp Stool DNA Kit (TIANGEN). Plasmids containing the target sequences of *E. faecalis* 16 S rRNA and TDC were prepared (Figure S1A,B). Then, 10‐fold serial dilutions of plasmid were used as the templates to make the standard curves for absolute quantification. The set of special primers is listed in Table S2. Quantitative PCR (qPCR) experiments were performed on 40 ng DNA in 20 μL reactions, following the amplification program: initial denaturation at 95°C for 15 min, 45 cycles of 95°C for 10 s, 58°C for 31 s, 72°C for 30 s, ended with a final extension at 72°C for 10 min. The absolute *E. faecalis* 16 S rRNA gene and TDC gene copies in fecal DNA samples were calculated based on the standard curves.

### 2.17 Determination of TDC in rat feces by ELISA

The rat feces were weighed and suspended in lysis buffer. After centrifugation at 800 *g* for 10 min, the supernatants were collected and added with lysozyme. The mixtures were centrifuged at 12,000 *g* for 20 min, and the supernatants were obtained for protein concentration determination and the detection of TDC level in rat feces using rat TDC ELISA kit (MM‐71824R1, MEIMIAN). The results are expressed as U/mg protein.

### 2.18 Preparation of PGF rats

Eighteen SD rats (300 g) were treated with the antibiotic mixture (200 mg/kg neomycin sulfate + 200 mg/kg ampicillin + 200 mg/kg streptomycin; Solarbio) twice a day for 7 days.^27^ The PGF state was confirmed by culturing fecal samples in nutrient medium under anaerobic condition; besides, the abundances of *E. faecalis* and TDC were also detected according to the qPCR method described above. Then rats were randomly divided into three groups, including (1) PGF group (treated with 0.5% CMC‐Na), (2) PGF + L‐dopa (60 mg/kg) group, and (3) PGF + L‐dopa (60 mg/kg) + PIP (20 mg/kg) group. The rats were given different treatments according to the groups once a day for 6 days. Meanwhile, the antibiotic administration (once a day) was maintained until Day 10. After the latest dosing, the blood and brain samples were collected, and the plasma L‐dopa level and striatal L‐dopa and DA contents were detected according to the above processing procedures and analysis methods.

### 2.19 *Enterococcus faecalis* transplantation

Eighteen SD rats (300 g) were pretreated with mixed antibiotics described above twice a day for 7 days. After verifying the PGF status through fecal sample culture, rats were randomly divided into three groups, which were (1) Transplanted group, (2) Transplanted + L‐dopa group, and (3) Transplanted + L‐dopa + PIP group. Rats were orally given *E. faecalis* (6 × 10^8^ CFU/day) from Day 7 to Day 10.^24^ Verified the colonization status through fecal sample culture and quantitative detection of *E. faecalis* and TDC. Rats were administered with L‐dopa or L‐dopa + PIP once a day from Day 7 to Day 13, then rat fecal samples were collected for qPCR analysis. After the latest dosing, the blood and brain samples were collected for the determination of L‐dopa and DA levels using the methods described above.

### 2.20 Statistical analysis

All data were analyzed and plotted by GraphPad Prism version 7.0. The datasets of behavior tests, quantification of striatal DA, and western blot assay in 6‐OHDA‐lesioned rats are presented as the means ± SEM. The results of other biological tests are presented as the means ± SD. Data were tested for normality through the Shapiro–Wilk test, and then the differences in data that exhibited normal distribution were analyzed using Student's *t*‐test and one‐way ANOVA. The data of AIMs in animal behavior tests did not exhibit the normal distribution, so Kruskal–Wallis test was used to analyze the data difference. Specific tests and statistical significance are noted in the legend. *p* Values < 0.05 were considered statistically significant.

## 3 RESULTS

### 3.1 PIP suppressed the intestinal bacterial L‐dopa conversion in vitro

We performed metagenomics analysis of intestinal microbiota in 6‐OHDA‐lesioned rats treated with PIP (20 mg/kg) for 6 weeks, and the result showed that PIP significantly reduced the relative abundance of *E. faecalis* (Figure 1B). To evaluate whether PIP could affect the conversion of L‐dopa by intestinal bacteria, we incubated PIP (10 and 20 μg/mL) and L‐dopa together with the intestinal bacteria of SD rats in vitro for 12 h and 24 h and demonstrated the inhibitory effect of PIP by calculating the conversion rate (CR) of L‐dopa in cultures with different treatments. The results showed that compared with L‐dopa alone, the CRs of L‐dopa in the cultures treated with L‐dopa + PIP (10 and 20 μg/mL) for 24 h were significantly decreased by 17.02% and 27.55%, respectively (Figure 1C). Our previous studies found that PIP has wide tissue distribution and can cross the blood–brain barrier.^28^ Therefore, we further incubated L‐dopa and PIP with blood, brain homogenate, and small intestine homogenate of SD rats, respectively, to investigate the effects of PIP on L‐dopa metabolism. The results showed that there was no significant difference in the CRs of L‐dopa between L‐dopa + PIP and L‐dopa alone in above incubation (Figure S2A–C), indicating that the intestinal bacteria might play a key role in the inhibitory effect of PIP on the conversion of L‐dopa.

TDC‐mediated decarboxylation shows considerable species dependence, and *E. faecalis* is the strain with the highest efficiency of decarboxylation.^14^, ^15^ We co‐incubated *E. faecalis* ATCC29212 with L‐dopa and various concentrations of PIP. As shown in Figure 1D, compared with L‐dopa alone, co‐treated with PIP (10 and 20 μg/mL) for 24 h could significantly suppress the metabolism of L‐dopa. The CRs of L‐dopa in L‐dopa + PIP groups were remarkably decreased by 17.22% and 32.01%. We tested the direct inhibitory effect of PIP on TDC by enzyme assay in vitro, and evaluated the activity of TDC by measuring the content of DA produced in the reaction system. The results showed that PIP exhibited a weak inhibitory effect toward TDC with a 20.95% decrease in TDC activity at the solubility limit of 250 μM (Figure S3). Considering that the level of TDC is another important factor affecting the ability of *E. faecalis* to convert L‐dopa,^29^, ^30^ we investigated the regulation of PIP on the gene expression and specific activity of TDC in *E. faecalis* ATCC29212 by RT–qPCR and Elisa. Remarkably, PIP reduced the expression and specific activity of TDC in *E. faecalis*, and the downregulation effects were notable when combined with L‐dopa (vs. L‐dopa; Figure 1E,F).

### 3.2 PIP combined with L‐dopa improved motor disorders and DA levels in 6‐OHDA‐lesioned rats

**FIGURE 2:**
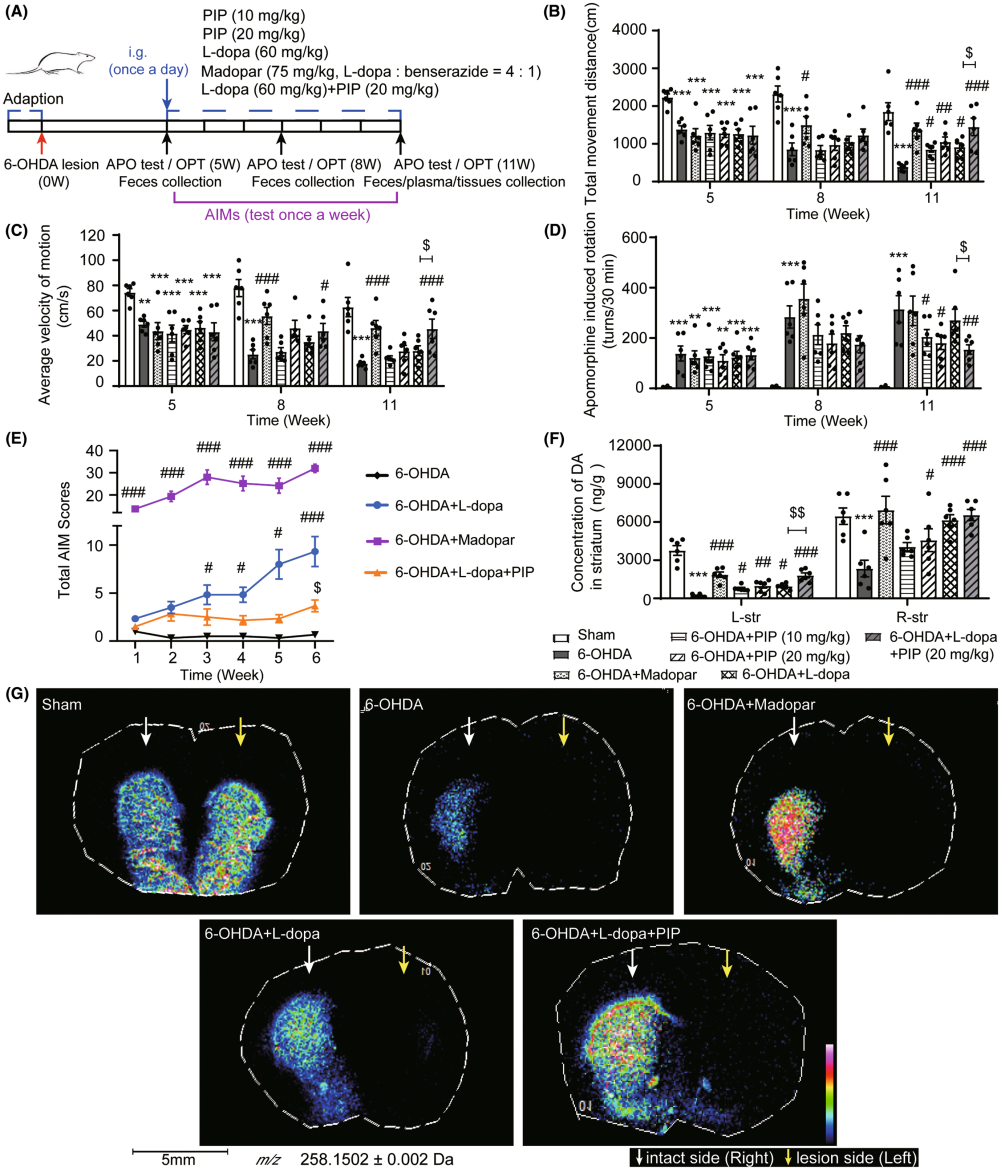
PIP combined with L‐dopa‐ameliorated 6‐OHDA‐induced motor disorders by improving brain DA levels in PD rats. The 6‐OHDA‐lesioned rats were administrated with L‐dopa (60 mg/kg), madopar (75 mg/kg, L‐dopa : benserazide = 4:1), PIP (10 mg/kg), PIP (20 mg/kg), and L‐dopa (60 mg/kg) + PIP (20 mg/kg) for 6 weeks, respectively. (A) Experiments scheme of 6‐OHDA‐lesioned rats. The total movement distance (B) and the average velocity of motion (C) of rats in the open field. (D) The number of rotations induced by APO. (E) Total AIM scores (Kruskal–Wallis test). (F) The content of DA in rat striatum was determined by HPLC‐ECD (L‐str: left striatum; R‐str: right striatum). Data are presented as mean ± SEM (*n* = 5–6). **p < 0.01, ***p < 0.001 versus sham group; ^#^ *p* < 0.05, ^##^ *p* < 0.01, ^###^ *p* < 0.001 versus 6‐OHDA group; ^$^ *p* < 0.05, ^$$^ *p* < 0.01, ^$$$^ *p* < 0.001 versus 6‐OHDA + L‐dopa group (one‐way ANOVA followed by a Fisher's LSD test). (G) The determination of relative abundance and distribution of DA by TMP‐TFB‐derivatized MALDI‐MS imaging method with a spatial resolution of 80 nm.

To support our data in vitro, we examined the synergistic effect of the combination of L‐dopa and PIP on 6‐OHDA‐lesioned rats (Figure 2A). Motor behavior was evaluated with the open‐field test, rotation assay, and AIMs rating. In the open‐field test, the movements of rats were significantly decreased after 6‐OHDA lesion (vs. sham). The L‐dopa administration could alleviate the bradykinesia in 6‐OHDA‐lesioned rats, whereas the co‐administration of L‐dopa + PIP showed more remarkable effects on improving the total distance (Figure 2B) and movement speed (Figure 2C) than L‐dopa alone. Similarly, in the rotation assay, L‐dopa + PIP abrogated motor deficits in 6‐OHDA‐lesioned rats by sustainably reversing the increase in rotation induced by APO, of which the efficacy was better than that of L‐dopa alone (Figure 2D). The long‐term administration of L‐dopa could induce dyskinesia (LID) in 6‐OHDA‐lesioned rats.^31^ By rating the AIMs of rats in different treatment groups, we observed notable LID in rats during different courses of madopar and L‐dopa administrations, whereas the rats treated with L‐dopa + PIP had no obvious LID (Figure 2E). The western blot assay of TH in rat SN further supported the results of behavior tests. The administration of PIP alone or in combination with L‐dopa could efficiently attenuate the lesions induced by 6‐OHDA on dopaminergic neurons (Figure S4).

HPLC‐ECD analysis showed that the DA levels in the striatum of 6‐OHDA‐lesioned rats were much lower than those in sham rats, which could be significantly increased by L‐dopa administration. Notably, L‐dopa combined with PIP could remarkably improve DA levels in rat lesioned striatum compared with L‐dopa alone (Figure 2F). To further assess this result, we used the TMP‐TFB‐derivatized MALDI‐FTICR‐MS imaging method to investigate the relative abundances and spatial distributions of DA in the striatum of rats. As shown in Figure 2G, compared with the sham rat, the relative abundance of DA in the lesioned (left side) striatum of the model rat was significantly decreased after 6‐OHDA injection. Moreover, the DA level in the non‐lesioned (right side) striatum of model rat was also significantly decreased due to the progressive loss of neurons. The L‐dopa and madopar treatments increased the DA level in the non‐lesioned side, respectively, and a more significant improvement was shown in rats with madopar administration. However, both of them had less effect on the DA level on the lesioned side, where no obvious DA signal was detected, and the imbalance of DA levels in the bilateral striatum may be a risk factor that triggered the development of LID.^32^ By contrast, the DA levels on both sides of striatum were significantly increased in 6‐OHDA‐lesioned rat after L‐dopa + PIP treatment (vs. 6‐OHDA + L‐dopa). These findings suggested that the combination of PIP and L‐dopa could better ameliorate 6‐OHDA‐induced motor disorders by improving brain DA level than L‐dopa alone.

### 3.3 PIP improved the availability of L‐dopa in 6‐OHDA‐lesioned rats

**FIGURE 3:**
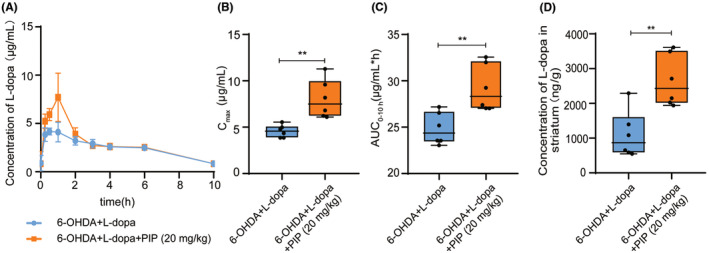
The availability of L‐dopa in 6‐OHDA‐lesioned rats was significantly improved by co‐administration with PIP. The 6‐OHDA‐lesioned rats were administrated with L‐dopa or L‐dopa + PIP, respectively, for 6 weeks. After the last administration, the plasma samples of rats were collected at different time points. The brain tissues were collected at 1 h after administration. The contents of L‐dopa were determined by HPLC‐FLD. (A) The concentration–time curves of L‐dopa in plasma of rats with different treatments. The *C*_max_ (B) and AUC_0‐10h_ (C) of L‐dopa in rats of different treatment groups. (D) The concentration of L‐dopa in rat‐lesioned striatum of different treatment groups. Data are presented as mean ± SD (*n* = 6). *p < 0.05, **p < 0.01, ***p < 0.001 versus 6‐OHDA + L‐dopa group (Student's *t*‐test).

We investigated the plasma pharmacokinetics of L‐dopa and the striatal L‐dopa contents in 6‐OHDA‐lesioned rats after co‐administrated with PIP and L‐dopa to further confirm the increasing level of DA in brain is related to the improvement of L‐dopa availability. The pharmacokinetics profiles of L‐dopa were presented in Figure 3A. In contrast with 6‐OHDA + L‐dopa group, the peak plasma concentration (*C*_max_) of L‐dopa in the 6‐OHDA + L‐dopa + PIP group was remarkably increased (Figure 3B). Moreover, PIP significantly increased the AUC_0‐10h_ of L‐dopa by 17.59% (Figure 3C). We further determined the level of L‐dopa in rat striatum 1 h after dosing, at which time the striatal L‐dopa concentration reaches the peak.^33^ Compared with the 6‐OHDA + L‐dopa group, the striatal L‐dopa level in 6‐OHDA + L‐dopa + PIP group remarkably increased by 2.43‐fold (Figure 3D). The results showed that PIP could effectively improve the availability of L‐dopa, thus increasing the contents of exogenous DA in the brain of 6‐OHDA‐lesioned rats. We hypothesized that this might be related to the inhibitory effect of PIP on the intestinal bacterial metabolism of L‐dopa.

### 3.4 PIP decreased the abundances of *Enterococcus faecalis* and TDC

**FIGURE 4:**
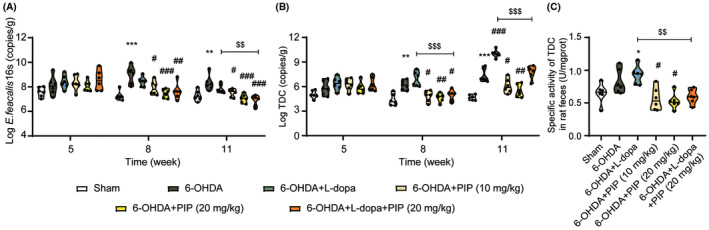
PIP decreased the abundances of *E. faecalis* and TDC in 6‐OHDA‐lesioned rats. The abundances of *E. faecalis* (A) and TDC (B) in rat feces at different time points after PIP administration were quantified by qPCR. (C) The specific activity of TDC in rat feces was detected by sandwich ELISA assay. Data are presented as mean ± SD (*n* = 6). *p < 0.05, **p < 0.01, ***p < 0.001 versus sham group; ^#^ *p* < 0.05, ^##^ *p* < 0.01, ^###^ *p* < 0.001 versus 6‐OHDA group; ^$$^ *p* < 0.01, ^$$$^ *p* < 0.001 versus 6‐OHDA + L‐dopa group (one‐way ANOVA followed by Tukey's multiple‐comparison test).

To demonstrate whether the improvement of oral L‐dopa availability is related to the effect of PIP on suppressing the intestinal bacterial L‐dopa conversion, we determined the *E. faecalis* and TDC levels in rat feces. The qPCR results showed that the abundances of *E. faecalis* were significantly increased in 6‐OHDA‐lesioned rats (vs. sham), and the administration of L‐dopa or madopar (Figure S5A) did not show a notable effect on decreasing the high levels of *E. faecalis* in model rats. In contrast, PIP could significantly reduce the *E. faecalis* abundance of 6‐OHDA‐lesioned rats in a dose‐dependent manner after 3 weeks of administration. Moreover, the down‐regulation effect of PIP was also remarkable when combined with L‐dopa, and the abundance of *E. faecalis* in 6‐OHDA + L‐dopa + PIP group was significantly lower than that of 6‐OHDA + L‐dopa group after 6 weeks of co‐administration (Figure 4A). TDC abundance plays a crucial role in regulating the L‐dopa availability.^14^, ^34^ As shown in Figure 4B, the expression of TDC in 6‐OHDA‐lesioned rats was significantly increased compared with that in sham rats, while the L‐dopa treatment further increased the TDC expression in model rats. Notably, PIP could effectively regulate the TDC expression of 6‐OHDA‐lesioned rats in a dose‐dependent manner, and a significantly suppressive effect could be observed after 3 weeks of administration. In addition, the combination of L‐dopa + PIP remarkably decreased the TDC expression in model rats, while the madopar administration showed no effect on that (vs. 6‐OHDA; Figure S5B). We further measured the specific activity of TDC in feces of rats after 6 weeks of administration using the ELISA kit, and the results were consistent with qPCR. PIP could significantly decrease the level of TDC in feces of model rats, and the suppressive effect was remarkable when combined with L‐dopa (vs. L‐dopa alone, Figure 4C).

### 3.5 The improvement effect of PIP on L‐dopa availability was microbiota dependent

**FIGURE 5:**
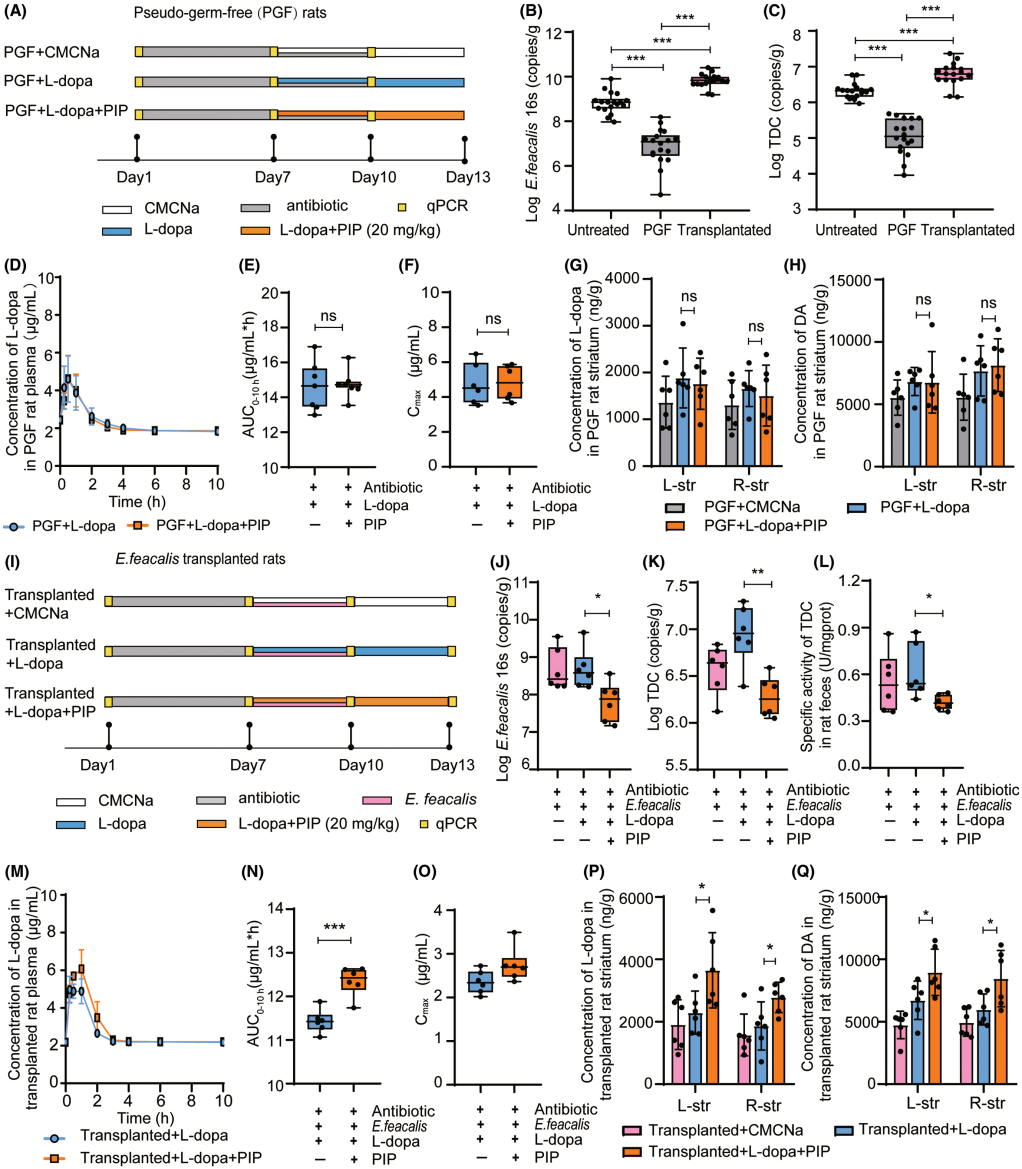
PIP increased L‐dopa availability in rats transplanted with *E. faecalis*. (A) The mode of treatments in PGF rats. The abundances of *E. faecalis* (B) and TDC (C) in rat feces before and after antibiotic treatment and *E. faecalis* transplantation. (D) The concentration–time curves of L‐dopa in PGF rats with different treatments. The *C*_max_ (E) and AUC_0‐10h_ (F) of L‐dopa in PGF rats with different treatments. The concentrations of L‐dopa (G) and DA (H) in PGF rat striatum of different treatment groups. (L‐str: left striatum; R‐str: right striatum) (I) The mode of treatments in rats transplanted with *E. faecalis*. The abundance of *E. faecalis* (J) and the gene expression (K) and specific activity of TDC (L) in transplanted rat feces after 1 week of administration. (M) The concentration–time curves of L‐dopa in transplanted rats with different treatments. The *C*_max_ (N) and AUC_0‐10h_ (O) of L‐dopa in transplanted rats with different treatments. The concentrations of L‐dopa (P) and DA (Q) in transplanted rat striatum of different treatment groups. Data are presented as mean ± SD (*n* = 6). *p < 0.05, **p < 0.01, ***p < 0.001 (Student's *t*‐test).

We established the PGF rats by oral antibiotics (Figure 5A) and verified the PGF status through fecal culture and qPCR detection.^24^, ^35^ The results showed that the intestinal colonies numbers (Figure S6) and *E. faecalis* and TDC abundances in feces of rats after antibiotic administration were remarkably decreased (Figure 5B,C). Then, we evaluated the effect of PIP on L‐dopa availability in PGF rats. The pharmacokinetics profiles of L‐dopa in PGF rats with different administrations are shown in Figure 5D. The AUC_0‐10h_ and *C*_max_ of L‐dopa in PGF rats treated with L‐dopa + PIP were not significantly different from those in rats treated with L‐dopa alone (Figure 5E,F). Further determined the levels of L‐dopa and DA in rat striatum by HPLC‐FLD and HPLC‐ECD, and the results showed no difference between the PGF + L‐dopa + PIP group and PGF + L‐dopa group (Figure 5G,H).

We further transplanted *E. faecalis* into the PGF rats (Figure 5I). The verification of colonial status showed that the intestinal colonies numbers (Figure S6) and *E. faecalis* and TDC abundances of rats after transplantation were remarkably increased (Figure 5B,C). The administration of L‐dopa had no significant effect on the abundances of *E. faecalis* in transplanted rats, moreover, L‐dopa induced the upregulation of TDC. In contrast, the combination of L‐dopa + PIP could remarkably decrease the abundance of *E. faecalis* and suppress the expression and specific activity of TDC (vs. L‐dopa alone; Figure 5J–L). We tested the effect of PIP on L‐dopa availability in transplanted rats. The pharmacokinetic curves are shown in Figure 5M. It was found that the AUC_0‐10h_ and *C*_max_ of L‐dopa in rats treated with L‐dopa + PIP were notably increased compared with those in rats treated with L‐dopa alone (Figure 5N,O). Besides, the levels of L‐dopa and DA in striatum of rats in the Transplanted + L‐dopa + PIP group were also significantly higher than those in the Transplanted + L‐dopa group (Figure 5P,Q). The improvement effect of PIP on L‐dopa availability was shown significantly in *E. faecalis*‐transplanted rats but blocked in PGF rats, which indicated the improvement effect of PIP on L‐dopa availability was microbiota dependent.

## 4 DISCUSSION

The complex interactions among “disease‐drug‐gut bacteria” become increasingly attractive, especially since recent studies have proved that the conversion of oral L‐dopa by intestinal bacterial TDC was a significant factor causing decline and inter‐individual variation in treatment efficacy among PD patients.^14^, ^15^, ^34^ In this study, we innovatively proposed that the combined administration of PIP and L‐dopa could increase the availability of L‐dopa as well as the brain DA level by reducing the abundances of *E. faecalis* and TDC, and finally, enhance the antiparkinsonian effect of L‐dopa in 6‐OHDA‐lesioned rats.

The currently symptomatic drug treatment of PD is still dependent on DA replacement therapy (DRT).^31^ However, the common therapy of AADCI combined with L‐dopa cannot inhibit bacterial TDC‐dependent L‐dopa conversion.^14^, ^15^ In addition, the DRT has less influence on the non‐motor symptoms and may induce LID, which is caused by the progressive loss of dopaminergic neurons and led to most disabilities in patients at the late stage of PD.^36^ A growing number of studies have suggested that traditional Chinese medicine (TCM) with multi‐component characteristics has synergistic effects on PD through multiple pathways based on multiple targets. For example, the combination of ginsenosides and L‐dopa can improve motor disorders and prevent LID in 6‐OHDA‐lesioned rats via neuroprotection.^37^ The combination of TCM and L‐dopa has become a new trend in the treatment of PD. However, the underlying mechanisms of the combined administrations have rarely been studied.

The previous chemical and pharmacological studies have shown that PLA, the multiple components with similar structures (Figure S7),^16^ are the major active constituents of *P. longum*, in which PIP with the highest content showed significant neuroprotective effects in a variety of PD animal models.^38^ Moreover, PIP with a long study history has been proven to have other excellent biological activities such as antibacterial, antioxidant, and antitumor.^39^, ^40^ Our metagenomics analysis of the gut microbiota in 6‐OHDA‐lesioned rats treated with PIP showed that the relative abundance of *E. faecalis*, the major TDC expression strain, was remarkably decreased. As the abundance of TDC has a significant impact on L‐dopa bioavailability,^14^, ^15^, ^34^ we investigated the effect of the combination of PIP and L‐dopa on 6‐OHDA‐lesioned rats and found that the motor deficits were dramatically improved compared with L‐dopa alone. The results of the pharmacokinetics study and the content determination of striatal L‐dopa showed that PIP remarkably increased the L‐dopa bioavailability and its level in the target tissue. We further determined the contents of DA in striatal homogenates by HPLC‐ECD and investigated the distribution and relative abundance of DA in the brain of 6‐OHDA‐lesioned rat by TMP‐TFB‐derivatized MALDI‐MS imaging. Both qualitative and quantitative results showed that the combination of PIP and L‐dopa could remarkably increase the brain DA level.

This result aroused our interest in exploring the synergistic mechanism of PIP combined with L‐dopa in ameliorating PD rats. In this study, we found that PIP could improve L‐dopa availability by suppressing intestinal bacterial L‐dopa metabolism. The abundance of *E. faecalis* detected by qPCR in feces of PD model rats was significantly increased compared with those in the sham rats. This result is also consistent with the clinical studies, the overgrowth of small intestinal bacteria caused by impaired intestinal motility in PD patients can lead to significant increase in the abundances of Bacilli (including the *E. faecalis*).^41^, ^42^, ^43^, ^44^ According to our metagenomics analysis, the administration of PIP could regulate the structure of intestinal flora and especially decrease the relative abundances of *E. faecalis* in PD rats. Here, we further confirmed that the combination of PIP and L‐dopa could regulate the abundance of *E. faecalis* in PD rats to normal levels. Actually, *E. faecalis* should not be excessively decreased considering it plays various roles in maintaining human intestinal homeostasis and mediating the metabolism of lipids and neurotransmitters in the intestine.^43^, ^45^, ^46^ More importantly, the level of TDC is pivotal to the efficiency of L‐dopa conversion by *E. faecalis*. We found that TDC was highly expressed in PD model rats and could be even further increased after the L‐dopa administration. A recently reported 2‐year longitudinal cohort study supported our results, which showed that the abundance of TDC in the intestine of PD patients increased rapidly compared with healthy controls. Moreover, the study also found that in addition to monoamine oxidase (MAO) inhibitors, L‐dopa and other adjuvant drugs (including AADCI and catechol‐O‐methyl transferase inhibitors) currently used in clinics play a positive role in increasing the abundance of TDC in the intestine of PD patient.^47^ PIP has been reported to have the potential to inhibit MAO activity.^48^ Our study found that the combination of PIP and L‐dopa could downregulate the gene expression and specific activity of TDC both in vitro and in vivo, thus effectively suppressing the abnormal hyper‐metabolism of L‐dopa by intestinal bacteria in PD model. Finally, we evaluated the effect of PIP on L‐dopa availability in PGF‐ and *E. faecalis*‐transplanted rats, and the results further validate the improvement of L‐dopa availability by PIP is mainly correlated with its regulation on intestinal bacteria, especially on *E. faecalis* TDC.

## 5 CONCLUSION

**FIGURE 6:**
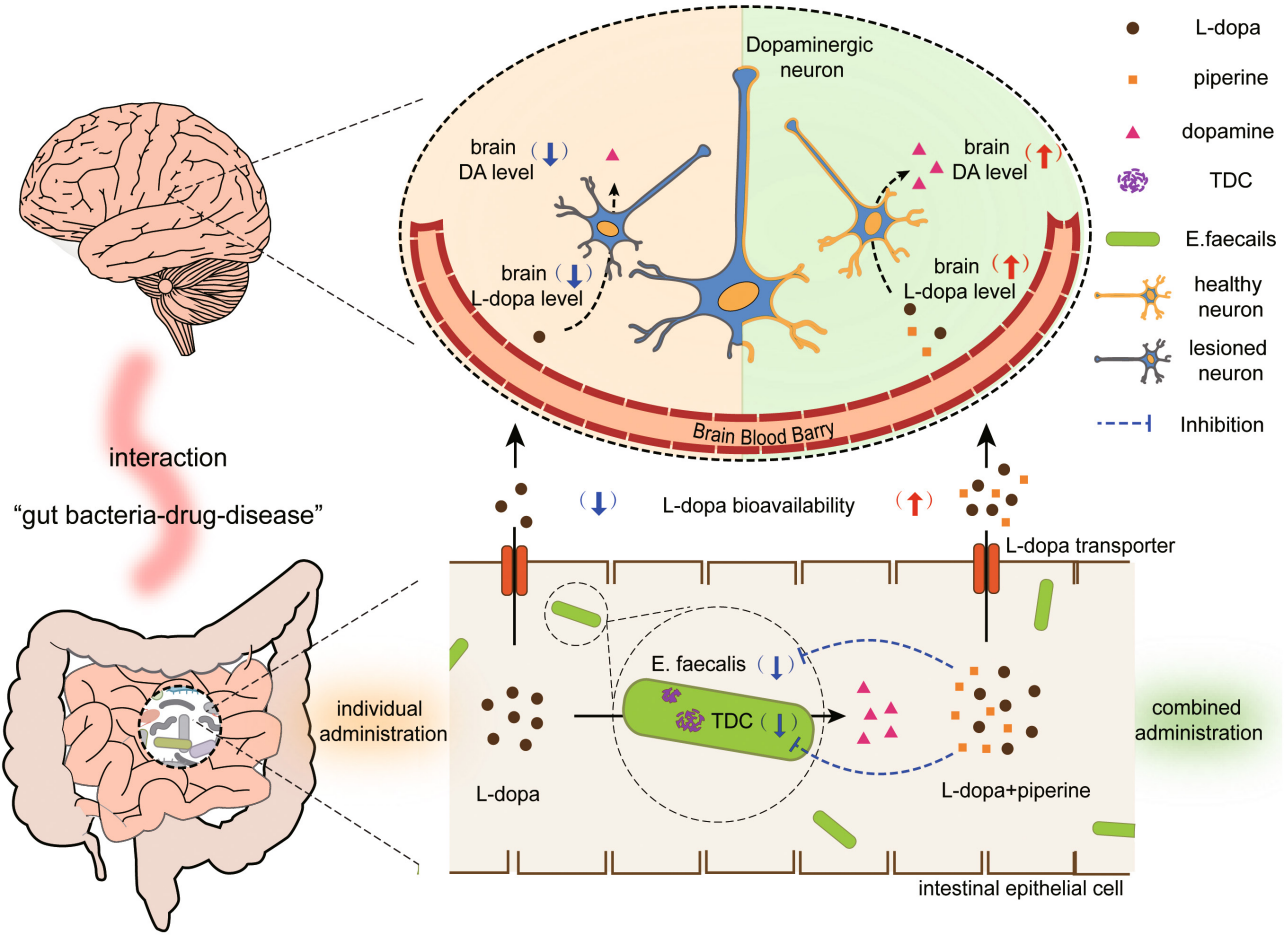
The combined administration of PIP and L‐dopa could improve the availability of L‐dopa and the brain DA level by suppressing the intestinal bacterial metabolism of L‐dopa by decreasing the abundances of *E. faecalis* and TDC.

In summary, the mechanism of the combination of PIP and L‐dopa improving the oral L‐dopa availability and brain DA level in 6‐OHDA‐lesioned rats may be related to the suppression of PIP on the intestinal bacterial metabolism of L‐dopa by reducing the high abundances of *E. faecalis* and TDC in model rats (Figure 6). The Piperaceae plants are rich in PIP, the fruit of which is widely used as a dietary flavor. It is necessary to apply the combined administration of PIP and L‐dopa on PD patients to further assess the effect on improving motor disorders and DA level in brain. Our study provides a new paradigm for the treatment of PD with integrated traditional Chinese and Western medicine, suggesting that PIP can be used as a potential adjuvant drug for PD treatment combined with L‐dopa.

## AUTHOR CONTRIBUTIONS

Xiaolu Hu and Xia Wu conceived and designed the study. Xiaolu Hu and Lan Yu performed most of the experiments and analyzed the data. XiaoXi Li, Yimeng Zhao, Lijuan Xiong, Jiaxuan Ai, and Qijun Chen helped with the animal experiments. Yatong Li and Xing Wang provided the software technical support. Yaonan Wang provided the MALDI‐MS imaging technical support. Xiaolu Hu and Xia Wu wrote the original manuscript that was reviewed by Xiaoqing Chen and Yinying Ba.

## FUNDING INFORMATION

This work was supported by the National Natural Science Foundation of China (no. 82173943, 81473333).

## CONFLICT OF INTEREST STATEMENT

The authors declare no competing interests.

## Supporting information


Appendix S1

Figure S4

## Data Availability

The metagenomics sequencing data that support the findings of this study have been deposited in the Genome Sequence Archive in BIG Data Center, Beijing Institute of Genomics (BIG), Chinese Academy of Sciences, under accession number CRA008435 that is publicly accessible at https://ngdc.cncb.ac.cn/gsa/.
