# Supplementary material for: Piperine improves levodopa availability in the 6‐OHDA‐lesioned rat model of Parkinson's disease by suppressing gut bacterial tyrosine decarboxylase
Source: CNS Neurosci Ther. 2023 Aug 1;30(2):e14383. doi: 10.1111/cns.14383 (PMC10848080; doi:10.1111/cns.14383)
Supplement: Supplementary file 1 — Appendix S1 [file CNS-30-e14383-s001.doc]

**Supplementary material for**

**Original article**

**Piperine improves levodopa availability in the 6-OHDA-lesioned rat model of Parkinson’s disease by suppressing gut bacterial tyrosine decarboxylase**


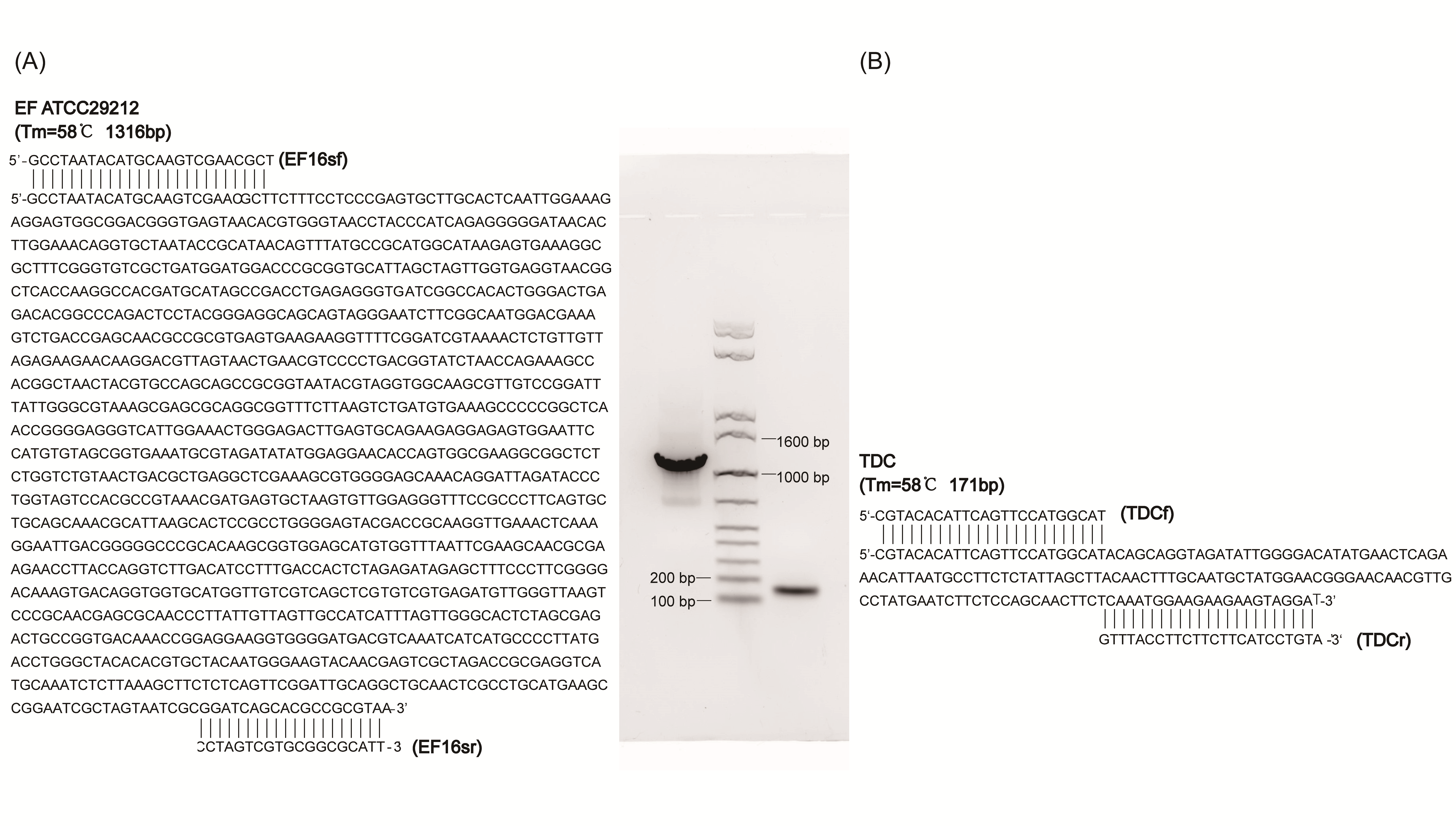


**Figure S1 Primers (EF16sf and EF16sr; TDCf and TDCr) targeting *E. faecalis* 16 S rRNA /TDC gene.** Targets of the EF16sf and EF16sr primers and the TDCf and TDCr primers are depicted for *E. faecalis* ATCC29212 with the corresponding agarose gel of PCR amplification of 1316 bp fragment of *E. faecalis* 16 S rRNA (A) and 171bp fragment of TDC (B).


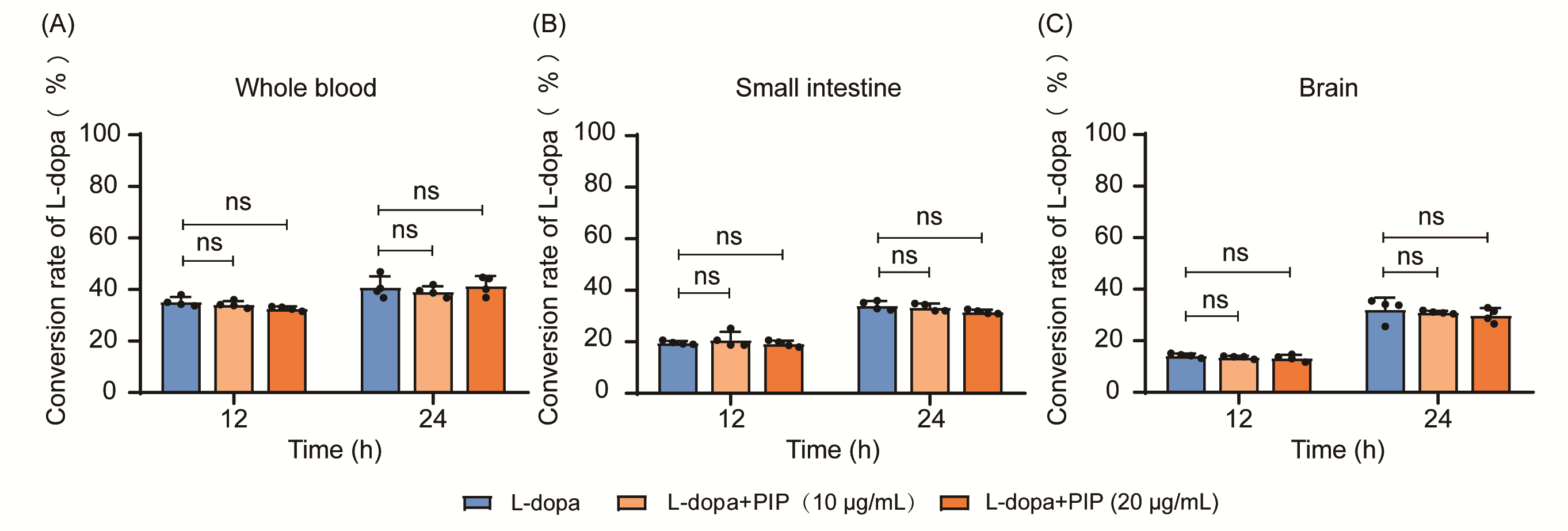


**Figure S2 PIP showed no influence on L-dopa conversion in blood, brain and small intestine tissues *in vitro*.** Rat whole blood, small intestine homogenate and brain homogenate were incubated with L-dopa (0.3 mM) or L-dopa (0.3mM) + PIP (10 or 20 μg/mL) for 12 h and 24 h, respectively. The levels of remaining L-dopa in systems were determined by HPLC-FLD. The conversion rate (CR) is calculated according to the formula which is “CR = (C_t_ – C_I_)/C_I_×100%”(C_t_: Remaining concentration of L-dopa at t h; C_I_: Initial concentration of L-dopa). (A) The CR of L-dopa in the blood *in vitro*. (B) The CR of L-dopa in the small intestine homogenate *in vitro*. (C) The CR of L-dopa in the brain homogenate *in vitro*. Data are presented as mean ± SD (n = 4). ns, *p*＞0.05 *vs* L-dopa group (One-way ANOVA followed by Tukey’s multiple comparison’s test).


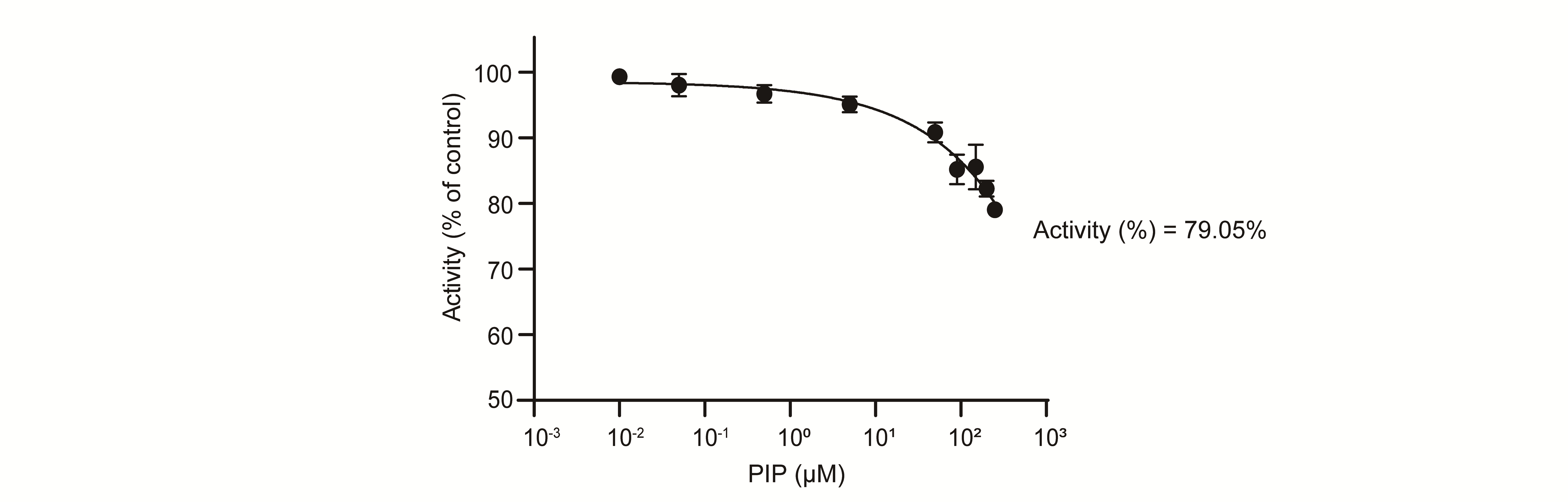


**Figure S3 The inhibitory effect of PIP on the activity of TDC in the enzyme assay.** Various concentrations (0.01-250 μM) of PIP were incubated with TDC and L-dopa, respectively, and the contents of DA were measured by HPLC-FLD. “Activity%” represents the content of DA relative to solvent control (0.1% DMSO). The activity of TDC decreased by 20.95% when treated with 250 μM PIP.


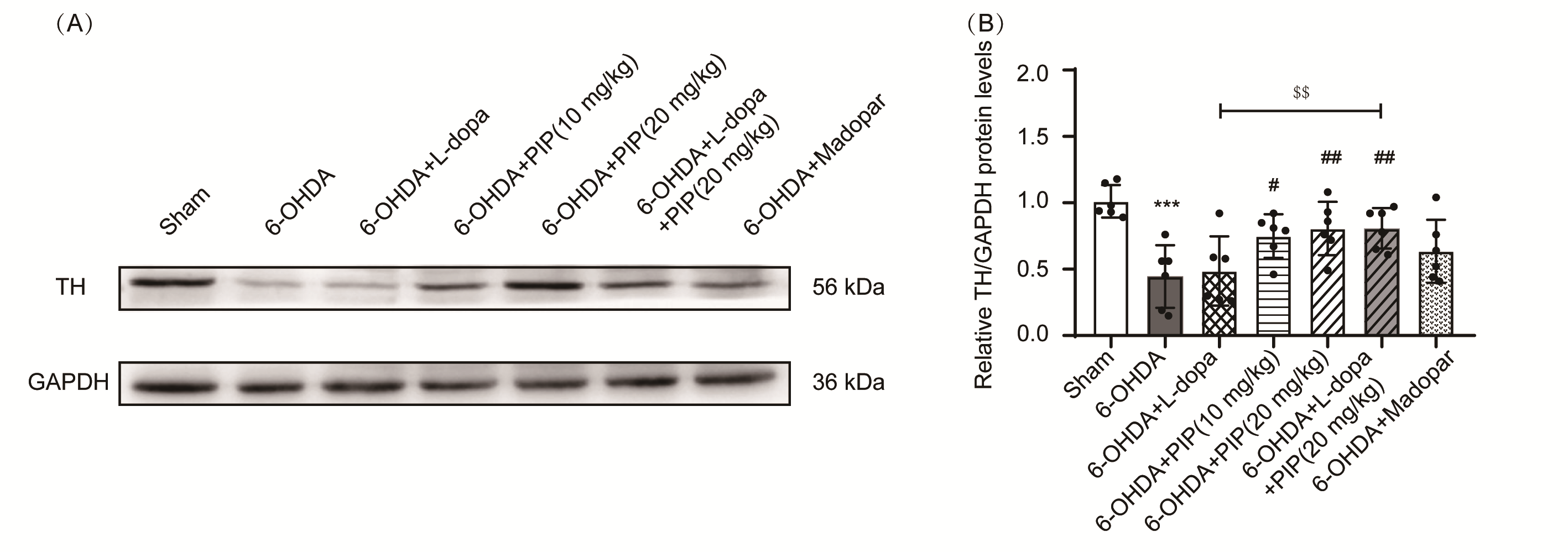


**Figure S4 The relative protein level of tyrosine hydroxylase (TH) in lesioned SN was assessed by western blot.** The rats were administrated with L-dopa (60 mg/kg), madopar (75 mg/kg, L-dopa: benserazide = 4: 1), PIP (10 mg/kg), PIP (20 mg/kg), L-dopa (60 mg/kg) + PIP (20 mg/kg) for 6 weeks, respectively. (A and B) Western blot analysis and quantification of the relative protein level of TH in the lesioned SN of rat, GAPDH was used as the internal control. Data are presented as mean ± SEM (n = 6). ^***^*p* < 0.001 vs Sham group; ^#^ *p* < 0.05, ^##^ *p* < 0.01 vs PD group; ^$$^ *p* < 0.01 vs L-dopa group (one-way ANOVA followed by a Fisher’s LSD test).


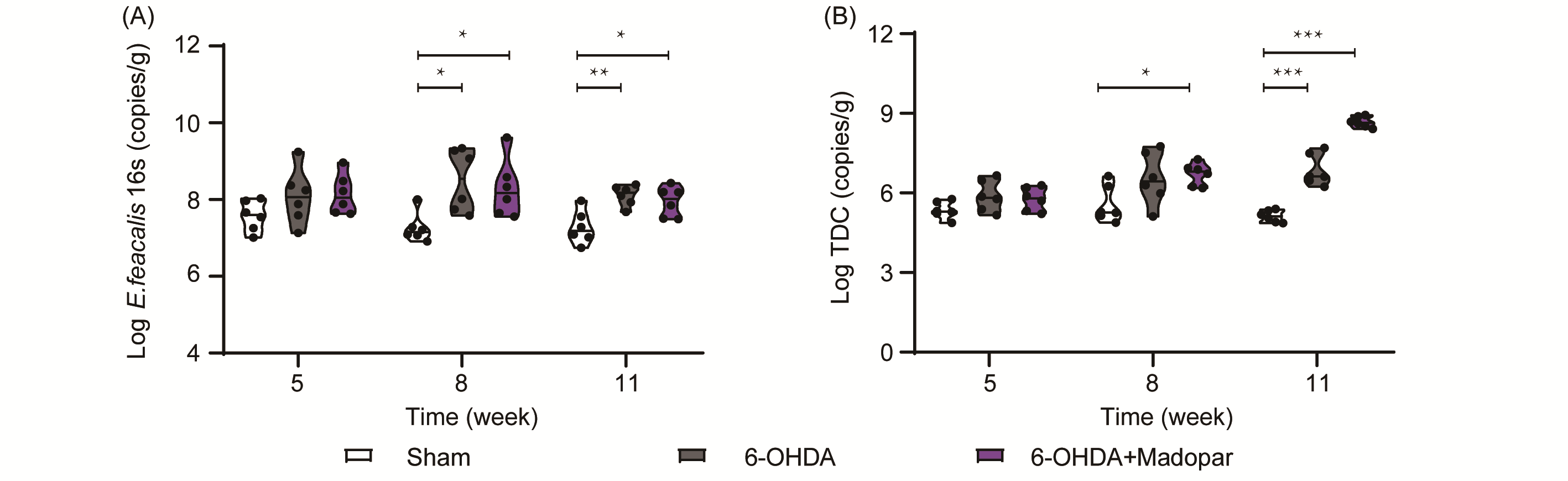


**Figure S5 Madopar showed no regulation effects on the abundances of *E. faecalis* and TDC.** The abundances of *E. faecalis* (A) and TDC (B) in rat feces at different time points after madopar administration were quantified by qPCR (n = 6). The data are presented as mean ± SD. ns, ^*^*p*＜0.05, ^**^*p*＜0.01, ^***^ *p* < 0.001 *vs* 6-OHDA group (One-way ANOVA followed by Tukey’s multiple comparison’s test).


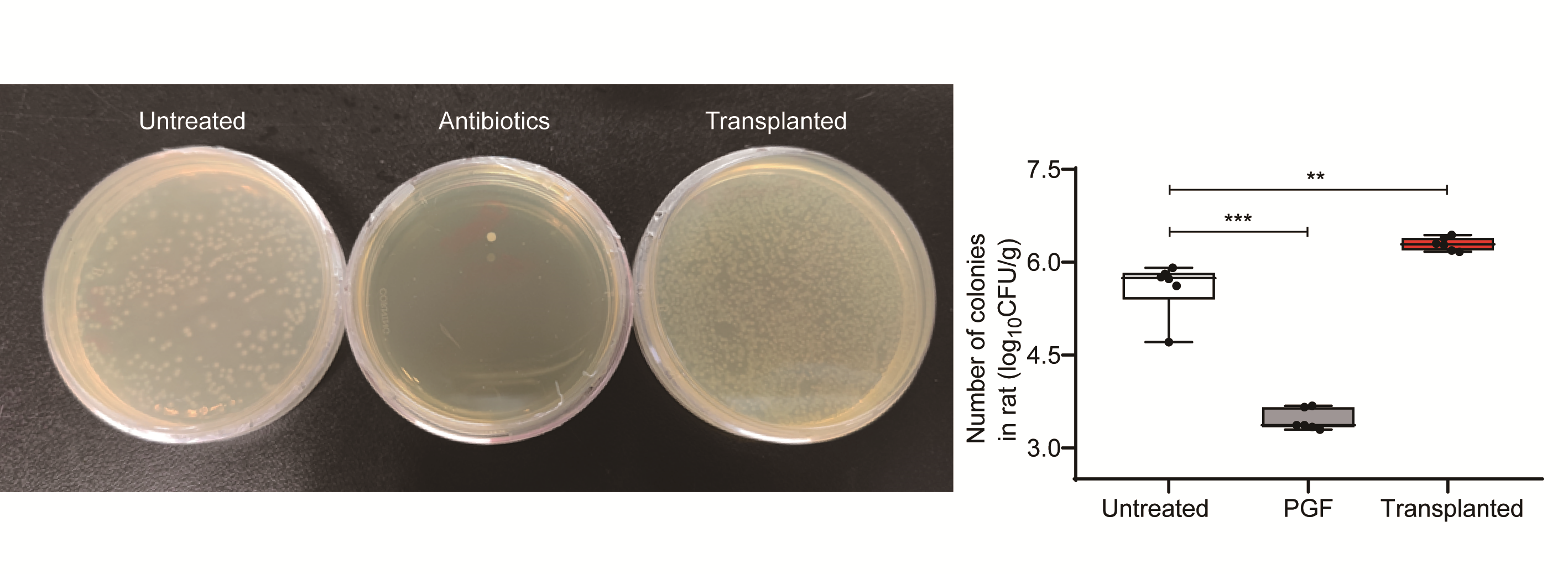


**Figure S6 The number of colonies of the rats treated with antibiotics and transplanted with *E. faecalis*.** Collected the fecal samples of rats with different treatments, suspended in sterile saline at a ratio of 1:20 (g/mL). After standing for 10 min, the supernatant was separated and diluted with saline, then coating and inoculating in the nutrient medium for 24 h, the number of colonies was calculated.


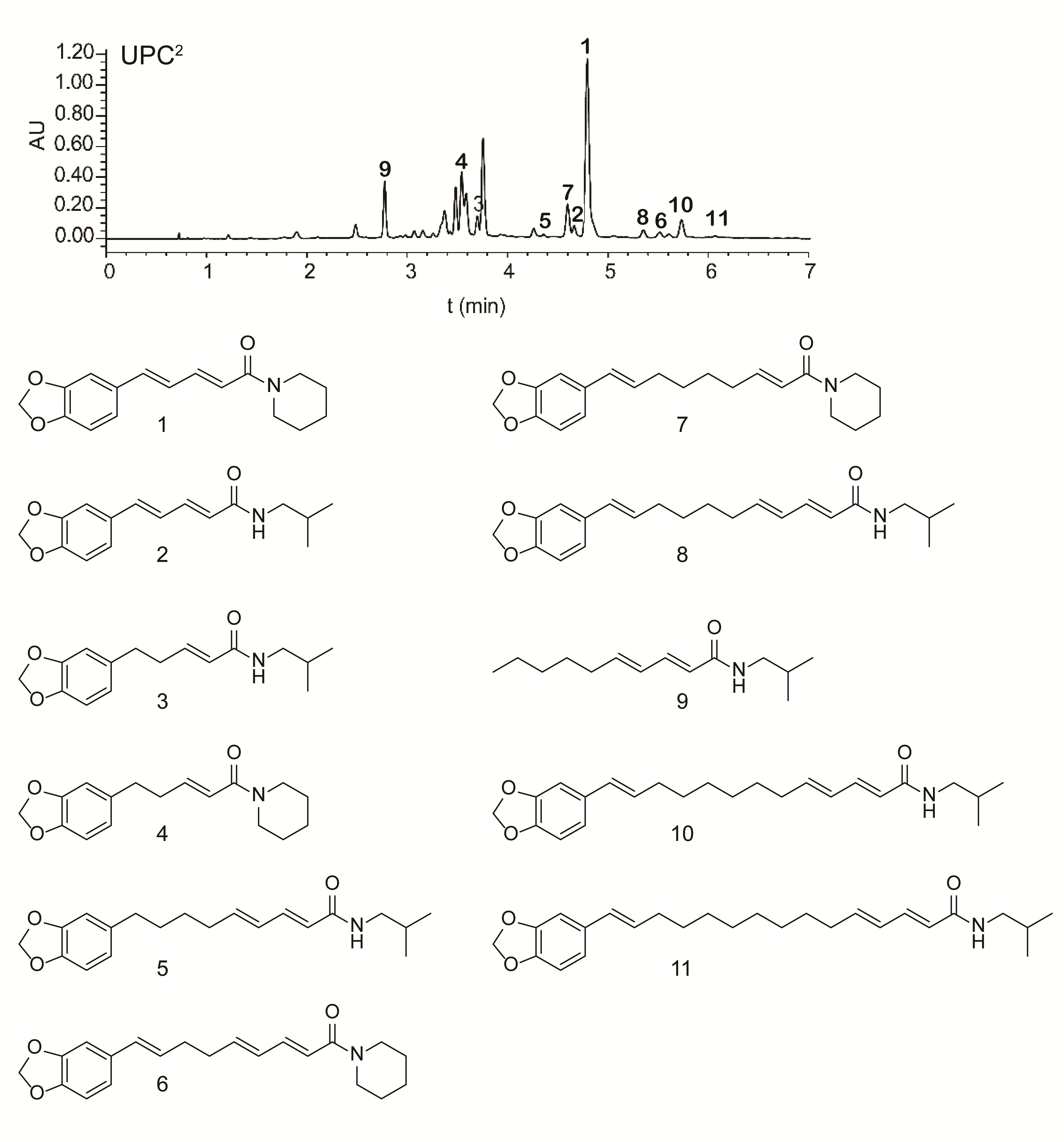


**Figure S7 UPC^2^-MS fingerprint of PLA.** (1) piperine, (2) piperlonguminine, (3) dihydropiperlonguminine, (4) piperanine, (5) pipercallosine, (6) dehydropipernonaline, (7) pipernonatine, (8) retrofractamide_B, (9) pellitorine, (10) guineensine, and (11) brachystamide_B.

**Table S1 The determination of the relative abundance of *E. faecalis* by metagenomics analysis**

| Gut microbiota | 6-OHDA | 6-OHDA+PIP (20 mg/kg) |
| --- | --- | --- |
| *Enterococcus_faecalis* | 0.0001148±0.000001242 | 0.00008392±0.000004562^*^ |

Data are presented as mean ± SD, n = 6. ^*^*p* <0.05 *vs* PD rats (Tow-tailed t test)

**Table S2 Primers used in quantitative by qPCR**

| Gene | Primer sequence (5’-3’) | Primer name | Reference |
| --- | --- | --- | --- |
| *E. faecalis* 16 S rRNA | GCCTAATACATGCAAGTCGAACGCT | EF16sf ^†^ | This study |
|  | TTACGCGGCGTGCTGATCC | EF16sr ^†^ |
|  | CGCTTCTTTCCTCCCGAGT | Faecalf ^‡^ | [1] |
|  | GCCATGCGGCATAAACTG | Faecalr ^‡^ |
| TDC | CGTACACATTCAGTTCCATGGCAT | TDCf ^†, ‡^ | [2] |
|  | ATGTCCTACTTCTTCTTCCATTTG | TDCr ^†，‡^ |
| recA | CAAGGCTTAGAGATTGCCGATG | recAf ^‡^ | [3] |
|  | ACGAGGAACTAACGCAGCAAC | racAr ^‡^ |

^†^ The primer used in conventional PCR

^‡^ The primer used in RT-qPCR

**Reference:**

[1] Ryu H, Henson M, Elk M, et al. Development of quantitative PCR assays targeting the 16S rRNA genes of Enterococcus spp. and their application to the identification of enterococcus species in environmental samples. *Appl Environ Microbiol*. 2013;79(1):196-204.

[2] Torriani S, Gatto V, Sembeni S, et al. Rapid detection and quantification of tyrosine decarboxylase gene (tdc) and its expression in gram-positive bacteria associated with fermented foods using PCR-based methods. *J Food Prot*. 2008;71(1):93-101.

[3] Perez M, Calles-Enríquez M, Nes I, et al. Tyramine biosynthesis is transcriptionally induced at low pH and improves the fitness of Enterococcus faecalis in acidic environments. *Appl Microbiol Biotechnol*. 2015;99(8):3547-3558.
