# Supplementary figures and images for: Piperine improves levodopa availability in the 6‐OHDA‐lesioned rat model of Parkinson's disease by suppressing gut bacterial tyrosine decarboxylase
Source: CNS Neurosci Ther. 2023 Aug 1;30(2):e14383. doi: 10.1111/cns.14383 (PMC10848080; doi:10.1111/cns.14383)

## Full unedited blots for Figure S4

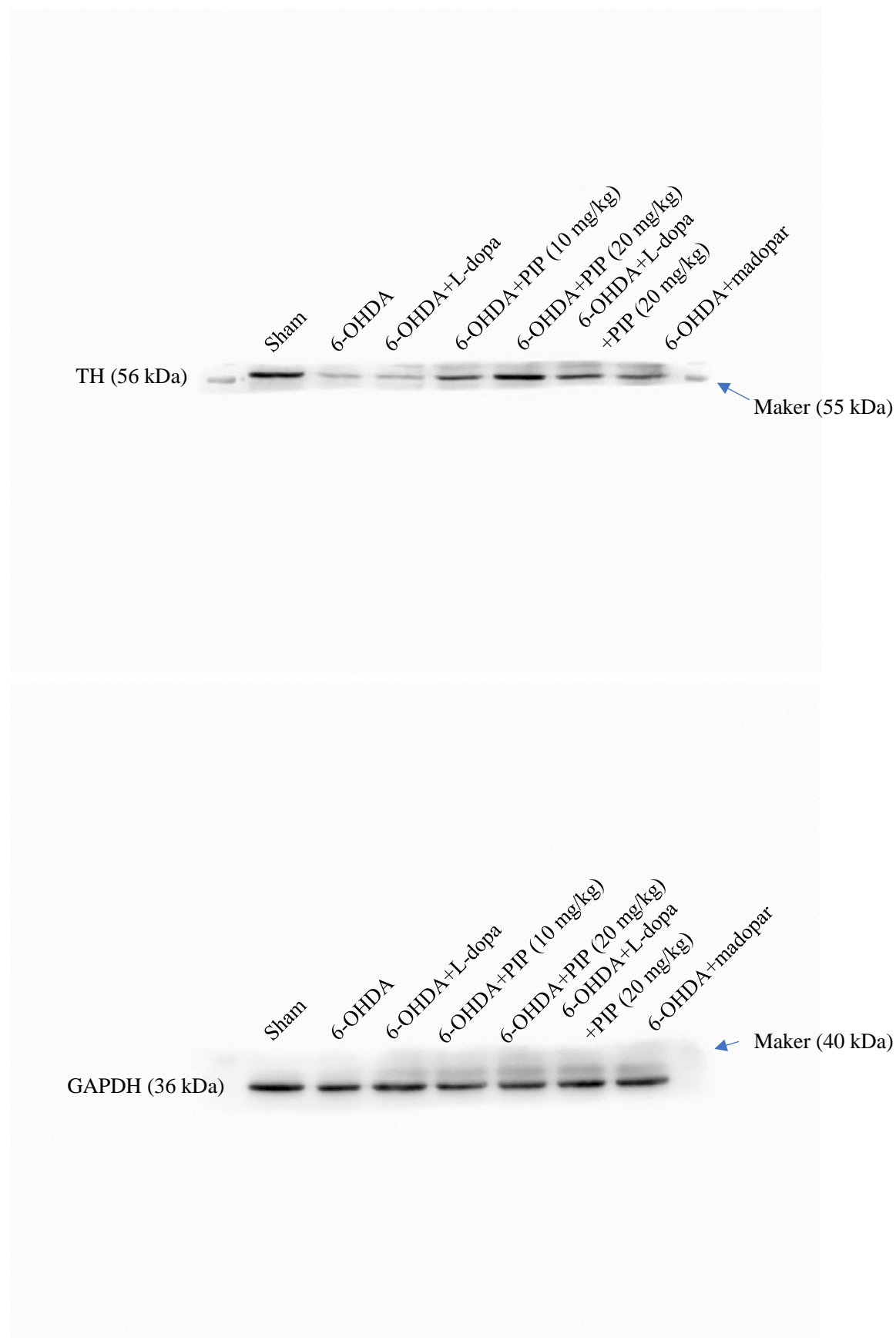

Supplement: Supplementary file 2 — Figure S4 [file CNS-30-e14383-s002.pdf]
